# Development of Standard Operating Protocols for the Optimization of *Cannabis*-Based Formulations for Medical Purposes

**DOI:** 10.3389/fphar.2019.00701

**Published:** 2019-06-25

**Authors:** Francesca Baratta, Marco Simiele, Irene Pignata, Lorenzo Ravetto Enri, Riccardo Torta, Anna De Luca, Massimo Collino, Antonio D’Avolio, Paola Brusa

**Affiliations:** ^1^Department of Drug Science and Technology, University of Turin, Turin, Italy; ^2^Laboratory of Clinical Pharmacology and Pharmacogenetics, Department of Medical Sciences, University of Turin, ASL Città Di Torino, Amedeo di Savoia Hospital, Turin, Italy; ^3^Academic Spin off CoQua Lab s.r.l, Turin, Italy; ^4^Department of Neurosciences “Rita Levi Montalcini,” University of Turin, Turin, Italy; ^5^University Hospital “Città della Salute e della Scienza di Torino,” Turin, Italy

**Keywords:** medical *Cannabis*, *Cannabis* oil, delta-9-tetrahydrocannabinol, cannabidiol, galenic preparations, standard operating protocols

## Abstract

Under current legislation in Italy, *Cannabis* for medical purposes may be administered orally in the form of decoction or *Cannabis* oil extract. The scientific literature reports a number of preparation methods, mainly for oils, but no study is available that compares thoroughly, from a technological viewpoint, the *Cannabis*-based formulations currently administered to patients. With this in mind, this research work aimed to carry out specific formulation studies to design standard operating procedures for the preparation and optimization of *Cannabis*-based galenic formulations. Both decoctions and oils were prepared under different operating conditions to identify the most efficient process for the production of formulations with a high concentration of decarboxylated delta-9-tetrahydrocannabinol (THC) and cannabidiol (CBD). Regarding *Cannabis* oil, a new procedure has been developed that allows significantly higher recovery rates for THC and CBD compared with those for water-based extraction methods (decoction) and those for oil-based methods currently in use. Moreover, based on the results, it is possible to affirm that the prescription of *Cannabis*-based decoctions should not be the recommended first-choice solution for therapy, considering the low concentration of THC and CBD and, consequently, the high volume of decoction that the patient would have to ingest.

## Introduction

In the past, *Cannabis* was widely used for its curative properties in traditional medicine. In the last century, it became the focus of attention for the abuse of its psychotropic effects. Consequently, the cultivation and sale of *Cannabis* were outlawed in many countries (Lafaye et al., 2017; Pisanti and Bifulco, 2019). However, in recent years, cannabinoids have seen a resurgence in consumption, in part, because of media attention and, in part, because of misplaced expectations of efficacy in some pathologies unsupported by scientific literature (Hill, 2015; Whiting et al., 2015).

To date, studies on the therapeutic efficacy of *Cannabis* in certain pathologies have yielded results that are, at best, contradictory and, generally, inconclusive given that the studies were carried out on inhomogeneous populations, used differing extraction processes, and administered differing dosages (Bar-Lev et al., 2018). Moreover, the experiments were performed without proper control procedures and were administered by different routes. These uncertainties stem in part from legislative restrictions that, over time, have severely hindered the performance of rigorous clinical studies under controlled and comparable conditions. The legalization of *Cannabis* for medical use can pave the way for the gathering of reliable clinical and epidemiological data—fundamental for a clear definition of the clinical efficacy and the inherent risks, in a medical environment, of *Cannabis*.

In this context, Italian legislation has recently relaxed regulations on the administration of medical *Cannabis* for a number of medical conditions; it is now possible to use medical *Cannabis* in pain therapy, in the treatment of chemotherapy- and radiotherapy-induced nausea and vomiting, and to stimulate appetite in cases of cachexia, anorexia, cancer patients or AIDS patients suffering from loss of appetite. It is also being used for other conditions such as glaucoma and Tourette’s syndrome. Moreover, the cultivation of *Cannabis* for medical use was recently authorized, and, since 2016, a variety of *Cannabis*, known as FM2, has been available from the Pharmaceutical Chemical Military Facility in Florence, grown under authorization from the Ministry of Health. This material is available as ground, dried flowering tops containing delta-9-tetrahydrocannabinol in concentrations ranging from 5% to 8% and cannabidiol from 7.5% to 12%. These percentages refer to the “total” content, that is, the sum of the components in acid form [delta-9-tetrahydrocannabinolic acid (THCA) and cannabidiolic acid (CBDA)] and decarboxylated form [delta-9-tetrahydrocannabinol (THC) and cannabidiol (CBD)] (President of the Italian Republic, 1990; Ministry of Health, 2015; Pharmaceutical Chemical Military Facility in Florence, 2019).

The phyto-complex of *Cannabis* plants is made up of more than 500 different constituents, of which a hundred or more belong to the cannabinoid class (Gould, 2015). Among the latter, even minor differences in structure can induce very different effects. The molecules of greatest interest from the point of view of their pharmacological activity are decarboxylated THC and decarboxylated CBD because they are more easily absorbed through the intestine (Grotenhermen, 2003). Therefore, the determination of the concentrations of these compounds in the preparations administered to patients is a fundamental prerequisite for therapeutic applications.

Current legislation in Italy allows *Cannabis* for medical purposes to be administered either orally or by inhaler. Regarding oral administration, according to the guidelines of the Ministry of Health, the first-choice pharmaceutical form is the decoction prepared in compliance with the official Ministry of Health procedure set out in the document “Recommendations for the prescribing doctor for the vegetable substance FM2 *Cannabis* flowering tops.” The inhaler route is only to be considered should the oral form not deliver the expected therapeutic effects or the patient’s doctor feels that it is more appropriate. Concerning oral administration, as well as the decoction, the current legislation allows for the administration of medical *Cannabis* as an oil extract (hereafter oil) provided that the oil content of active ingredients has been titrated by means of specific instrumentation (liquid or gas chromatography coupled with mass spectroscopy) (Ministry of Health, 2017;Ministry of Health, 2015).

The administration of titrated formulations, with a known quantity of active ingredient, obviously allows for a more uniform therapy and optimization of the risks/benefits. On this point, it is important to stress that although a number of preparation methods have been reported in scientific literature (Romano and Hazekamp, 2013; Citti et al., 2016; Società Italiana Farmacisti Preparatori, 2016), especially for oils, there is a lack of exhaustive comparative studies that examine from a technological aspect the *Cannabis*-based galenic formulation procedures currently in use for therapy.

In light of the above, the aim of this present work was to conduct specific formulation studies to design standard operating procedures for the preparation and optimization of *Cannabis*-based galenic formulations conforming with current health regulations. In particular, regarding the decoctions, the aims were to evaluate if concentrations were similar between preparations with a fixed ratio of *Cannabis* plant weight to solvent volume and, if it were possible, changing operating procedures to enrich the decoctions in terms of cannabinoid content. Concerning the oil, the principal aim was to evaluate how to set operating conditions to have the highest content of decarboxylated cannabinoids.

The application of a defined operating procedure, which produces reproducible results, is of particular relevance in obtaining standardized products. In particular, this is important in view of conducting further studies designed to optimize the administration of *Cannabis* for medical use based on patient characteristics. Furthermore, the application of standard procedures is important in view of performing clinical trials to evaluate the real correlation between cannabinoids and clinical outcomes in the real clinical practice.

## Materials and Methods

### Materials for Galenic Preparations

All of the preparations described below were based on flowering tops from type FM2 *Cannabis* purchased from the Pharmaceutical Chemicals Military Facility in Florence. The titrated concentrations of active compounds in the unprocessed material (May 2017) were 0.40% ± 0.02% for THC, 5.74% ± 0.18% for THCA, 0.29% ± 0.03% for CBD, and 8.70% ± 0.17% for CBDA. Consequently, the total THC content, calculated using the formula %THC tot = %THC + (0.877 × %THCA), was 5.43% ± 0.15% and the total CBD content, calculated using the formula %CBD tot = %CBD + (0.877 × %CBDA), was 7.92% ± 0.18%. The formulae adjusted for the differing molecular weights of the cannabinoid and carboxylic conjugative components of each cannabinoid. Indeed, the decarboxylated form has a lower molecular weight than that of the carboxylated one because of the loss of a CO_2_ molecule. The ratio between the two molecular weights is 0.877 (Pacifici et al., 2017).

At the end of the experimentation period (June 2018), the titrated quantities of the active constituents in the plant material, calculated using the same equations, were reassessed and were 2.54% ± 0.33% for THC, 2.97% ± 0.41% for THCA, 1.71% ± 0.26% for CBD, and 6.29% ± 0.72% for CBDA. It follows that the total THC was equal to 5.14% ± 0.69% and total CBD was 7.23% ± 0.95%. The other materials used for producing the preparations (olive oil and depured water) were purchased from a pharmaceutical supplies company (Farmalabor s.r.l., Canosa di Puglia, Bari, Italy) and complied with the relevant monograph of the European Pharmacopoeia (Eur.Ph).

Before being used for the formulations described below, the *Cannabis* plant material was ground for 60 s to produce a uniform blend.

### Reagents and Materials for Quantitative Analysis

Olive oil (pharmaceutical grade), CBD, cannabinol (CBN), CBDA, cannabidiol-d3 (CBD-d3), THC, (-)-delta-9-tetrahydrocannabinol-d3 (THC-d3), and isopropanol LC-MS grade were purchased from Sigma-Aldrich (Milan, Italy). THCA was purchased from LGC (Milan, Italy). Acetonitrile LC-MS grade was purchased from VWR (Milan, Italy).

HPLC-grade water was produced with Elix-coupled with Synergy-UV water purification system (Merck Millipore, Milan, Italy).

### Preparation of the Decoction

The official procedure issued by the Italian Ministry of Health specifies that a decoction of FM2 *Cannabis* must be based on a mixture of *Cannabis* FM2 plant material and cold water in a weight-to-volume ratio of 1:1 (mg/ml). It advises against using a quantity of water less than 100 ml. The *Cannabis* plant material is added to the cold water; the water is brought to boil and allowed to simmer on low heat for 15 min in a covered vessel. The recommended maximum decoction time is 30 min, and the mix should be stirred regularly. The decoction is allowed to cool for 15 min, then stirred and filtered. The residue trapped in the filter must be pressed with a spoon to recover as much liquid as possible and strengthen the final solution (Ministry of Health, 2017).

In accordance with the ministerial procedure, batches of decoction were prepared with a weight-to-volume ratio between plant material and solvent of 1:1 (mg/ml) (**Table 1**, Type A Decoctions).

**Table 1:** Types of decoction prepared.

Type of decoction	Ratio of *Cannabis* (mg)/solvent (ml)	Quantity of *Cannabis* (mg)	Volume of water (ml)
A-1	1:1	100	100
A-2	1:1	200	200
A-3	1:1	300	300
B-1	2:1	200	100
B-2	3:1	300	100
B-3	4:1	400	100
B-4	5:1	500	100
B-5	10:1	1,000	100

In addition, further batches were prepared in compliance with the ministerial procedure (decoction and filtration) but with a modified weight-to-volume ratio between *Cannabis* FM2 flowering tops and solvent, namely, batches of decoctions with weight-to-volume ratios ranging from 2:1 to 10:1 (mg/ml) (**Table 1**, Type B Decoctions).

### Common Methods for Preparing *Cannabis* Oils

Regarding *Cannabis* oils, there are, at present, three common methods for the preparation of *Cannabis*-based galenic preparations. The first two methods, which we shall call “RH” (Romano and Hazekamp, 2013) and “CC” (Citti et al., 2016), respectively, instruct that the *Cannabis* is finely chopped beforehand and then mixed with olive oil. The mixture is then heated (2 h immersed in a boiling water bath according to method RH; 2 h at 110°C according to method CC). The solution is then filtered to obtain the oily extract.

The third method, hereafter “SF” (Società Italiana Farmacisti Preparatori, 2016), instructs that the *Cannabis* is chopped finely and preheated at 115°C for 40 min. Thereafter, the *Cannabis* is added to the olive oil and further ground with a turbo emulsifier for 3 min. The mixture of olive oil and *Cannabis* is heated in a water bath of boiling water for 40 min and then filtered. Butyloxytoluene (BHT) is added to 0.02%.

All of the above methods are designed for a *Cannabis* plant weight–to–solvent volume ratio (mg/ml) of 100:1.

In the analysis of the experimental quantities of active ingredients that it is possible to recover from Type FM2 *Cannabis* plant using the three experimental methods above, the results were as follows:

- RH: 0.17% ± 0.09% THC, 0.05% ± 0.03% CBD, 0.18% ± 0.06% THCA, 0.47% ± 0.10% CBDA
- CC: 0.24% ± 0.08% THC, 0.23% ± 0.11% CBD, 0.24% ± 0.14% THCA, 0.67% ± 0.25% CBDA
- SF: 0.37% ± 0.08% THC, 0.70% ± 0.19% CBD, 0.07% ± 0.07% THCA, 0.15% ± 0.09% CBDA (Roda et al., 2018)

The most efficient method according to the results appears to be the SF procedure, which has a maximum titrated content of THC of 0.37% ± 0.08% (3.38 mg/ml) and a titrated CBD concentration of 0.70% ± 0.19% (6.40 mg/ml).

The commonly used extraction procedures to recover THC and CBD in oil form are not exhaustive. Therefore, the amount of THC and CBD present in the final preparations is lower than the declared one in the flowering tops.

### Preparation of Oils for the Study

To prepare the *Cannabis* oil batches for this study, a defined weight of type FM2 *Cannabis* was added to a determined volume of olive oil (mg/ml) in a ratio of 100:1 or 200:1. Batches of these oils were then heated in a water bath at 100°C with a magnetic stirrer for 30, 60, and 120 min, respectively. The oil was then filtered using a manual press through a filter of cotton or hydrophilic cotton gauze.

Before being added to the olive oil, some samples of the *Cannabis* material had been spread in a thin layer (maximum 5 mm, preferably 1–2 mm) and placed in an oven at 140°C for 30 min or at 115°C for 40 min. Other samples were added to the olive oil without preheating.

The temperatures applied were chosen for the following reasons: 115°C is near the decarboxylation reaction temperature for cannabinoids, which mainly takes place around 110°C, whereas 140°C was chosen as it is near the evaporation point of THC (145°C) (Wang et al., 2016).

The volumes of the prepared batches were between 5 and 100 ml.

**Table 2** lists the operating conditions applied during the preparation of the oils.

**Table 2:** Type of oil prepared.

Method	Ratio of *Cannabis* (mg)/solvent (ml)	Time in boiling water bath	Preheating temperature of the flowering tops	Time of preheating (min)
α-1	100:1	120	No preheating	/
α-2	100:1	120	115°C	40
α-3	100:1	30	140°C	30
α-4	100:1	60	140°C	30
α-5	100:1	120	140°C	30
β-1	200:1	120	No preheating	/
β-2	200:1	120	115°C	40
β-3	200:1	30	140°C	30
β-4	200:1	60	140°C	30
β-5	200:1	120	140°C	30

### Analytical Methods

Chromatographic analysis (Carcieri et al., 2018) was performed by Acquity^®^ UPLC system coupled with a TQD mass spectrometer (Waters, Milan, Italy). The chromatographic separation was carried out using an Acquity UPLC HSS T3 column (2.1 × 30 mm, 1.8 µm) (Waters, Milan, Italy) at a constant 30°C. The chromatographic separation was obtained by a gradient of mobile phases A (acetonitrile and water in a ratio of 70:30 + 0.05% formic acid) and B (isopropanol and acetonitrile in a ratio of 80:30 + 0.05% formic acid) at a flow rate of 0.4 ml/min. The initial condition of the gradient was 100% solution A; after 3.5 min, the mobile phase was brought to 100% solution B and kept there for 1.5 min; then the column was reequilibrated to the initial condition for 1 min (total time, 6 min). The autosampler was kept at 10°C, and the injection volume was 10 µl. Data acquisition, data processing, and system control were managed by MassLynx software (Waters, Milan, Italy). The mass spectrometer coupled to the UPLC system was set in positive ionization mode (ESI+) with a capillary voltage of 3.5 kV, a source temperature of 150°C, and a desolvation temperature of 400°C. The flow rate of the nitrogen was 800 L/h for the desolvation, and the cone flow rate was 60 L/h.

Ion monitoring was performed in multiple reaction modes, with the mass transitions and collision energies (CEs) as reported here: CBD 315.14 → 193.04, CE 25; CBD-d3 318.10 → 196.14, CE 25; THC 315.11 → 193.05, CE 25; THC-d3 318.19 → 196.12, CE 25; CBDA 359.15 → 219.07, CE 30; THCA 359.13 → 219.11, CEC 30; CBN 311.15 → 223.10, CE 20.

All the standard cannabinoid solutions necessary to create the calibration curve were diluted to concentrations between 1,250 ng/ml and 5 ng/ml. CBD-d3 and THC-d3 were used as internal standards.

All of the samples to be analyzed were diluted with isopropanol to obtain a final concentration suitable for the range of the calibration curve.

### Statistical Methods

The following values for each constituent of interest were calculated for the samples prepared according to the methods described in **Tables 1** and **2** (Average concentration, the relative standard deviation, and the maximum and minimum quantities found in a specific quantity of finished product).

The data distribution was evaluated by the Shapiro–Wilk test. For the data with a normal distribution, comparisons between proportions and mean values were performed using t-test or ANOVA test; for the data with a non-normal distribution, the Wilcoxon rank-sum test or Kruskal–Wallis test was used instead.

To evaluate the variation of the final concentration of THC in the decoctions, linear regression was used. The dependent variable was THC concentration, and the independent variable was the initial amount of *Cannabis* used. The same analysis was performed to evaluate the variation of the final concentration of CBD in the decoctions.

The level of significance was fixed at 0.05; CI at 95%. Statistical analysis was performed with Stata^®^14 (StataCorp. 2015. Stata Statistical Software: Release 14. College Station, TX: StataCorp LP).

## Results

### Decoctions

**Table 3** lists the quantities of THC, THCA, CBD, CBDA, and CBN found in the decoctions prepared using the procedures in **Table 1**. An analysis was performed on 146 samples of decoctions subdivided by the different preparation methods as reported in **Table 1**. Regarding decoctions prepared with methods A (A-1, A-2, A-3), the method with which was prepared the greatest number of samples was the method A-1. This corresponds to the one that provides the minimum dose of decoction for therapeutic purposes following the procedure approved by the Italian Ministry of Health. A-2 and A-3 methods were performed to confirm data when solvent volumes and *Cannabis* amounts, respectively, increase.

**Table 3:** Quantity of active constituent in the decoctions.

Method	Active molecule	Number of samples analyzed	Average concentration of active molecule (mg/100 ml)	Standard deviation	Concentration of active molecule: minimum value (mg/100 ml)	Concentration of active molecule: maximum value (mg/100 ml)
A-1	THC	39	0.87	0.41	0.26	2.47
	CBD		1.07	0.43	0.27	2.42
	THCA		1.81	0.55	0.88	3.09
	CBDA		4.83	1.07	2.59	7.71
	CBN		0.02	0.03	0.00	0.15
A-2	THC	6	0.72	0.15	0.53	0.88
	CBD		1.01	0.14	0.86	1.24
	THCA		1.81	0.89	0.58	2.52
	CBDA		5.18	0.91	3.87	6.38
	CBN		N.D.	N.D.	N.D.	N.D.
A-3	THC	6	0.83	0.26	0.59	1.31
	CBD		1.09	0.26	0.90	1.62
	THCA		1.91	0.78	0.92	2.60
	CBDA		5.36	0.63	4.29	6.12
	CBN		N.D.	N.D.	N.D.	N.D.
B-1	THC	4	1.33	0.24	1.08	1.54
	CBD		1.42	0.31	1.01	1.65
	THCA		2.92	0.44	2.45	3.44
	CBDA		9.58	0.84	8.88	10.61
	CBN		N.D.	N.D.	N.D.	N.D.
B-2	THC	4	2.44	0.43	1.81	2.80
	CBD		2.04	0.46	1.36	2.39
	THCA		2.95	0.58	2.13	3.37
	CBDA		11.12	2.72	7.13	13.03
	CBN		N.D.	N.D.	N.D.	N.D.
B-3	THC	4	3.86	0.35	3.61	4.36
	CBD		3.12	0.22	2.79	3.27
	THCA		3.52	0.03	3.49	3.57
	CBDA		13.82	0.23	13.66	14.16
	CBN		N.D.	N.D.	N.D.	N.D.
B-4	THC	38	6.24	1.32	3.10	8.55
	CBD		6.01	1.65	2.42	8.65
	THCA		4.04	1.14	1.79	7.15
	CBDA		14.44	3.08	7.64	22.62
	CBN		0.22	0.05	0.08	0.30
B-5	THC	45	13.81	3.89	6.68	20.30
	CBD		14.44	4.51	5.02	20.68
	THCA		5.14	1.43	1.75	8.24
	CBDA		21.37	4.09	11.66	30.22
	CBN		0.50	0.18	0.14	0.81

Based on the results obtained, it is possible to affirm that the methods A-1 to A-3, where the ratio of *Cannabis* plant weight to solvent volume was maintained constantly (1:1) and for identical volumes of final product, there is no significant difference in the quantity of active molecules extracted (p > 0.05). However, regarding the methods B-1 to B-5, a significant difference in the quantity of active molecule extracted was found. For these methods, it was possible to observe that, given an identical volume of solvent used, the increase in raw material added leads to an increase in the quantities of both THC and CBD extracted. The linear regression analysis highlighted an average increase in THC of 0.016 mg/100 ml (p < 0.001) and an average rise in CBD levels of 0.017 mg/100 ml (p < 0.0001) for each 1 mg increase in *Cannabis* plant material added.

For all the decoctions prepared according to the methods A-1 to B-2, regardless of the ratio of *Cannabis* plant material weight to solvent volume used, the acidic cannabinoids were present in greater concentrations than the decarboxylated forms. For the decoctions prepared with methods B-3 to B-5, with respect to THC and THCA, the ratio changed: the decarboxylated forms were more present than the acidic ones. Considering the average content of active constituents in the decoctions prepared following methods A-1 to A-3, the average proportion of acidic forms to carboxylated forms is similar for all samples and, on average, equal to 2.30 ± 0.218 for THC and 4.85 ± 0.315 for CBD. Regarding the decoctions prepared with methods B-1 to B-5 and considering the average content of active constituents, the proportions between the acidic forms and the decarboxylated forms diminish with the increase in the quantity of the plant form of the drug used: from a maximum of 2.19 for THC and 6.75 for CBD for method B-1 to a minimum of 0.37 for THC and 1.48 for CBD for method B-5.

CBN, a degradation product of THC, is present in minimal quantities in the samples of decoctions. The greatest quantities are present in decoctions prepared with methods B-4 and B-5, in which the highest amount of *Cannabis* flowering tops was used.

### Oils

**Figure 1:**
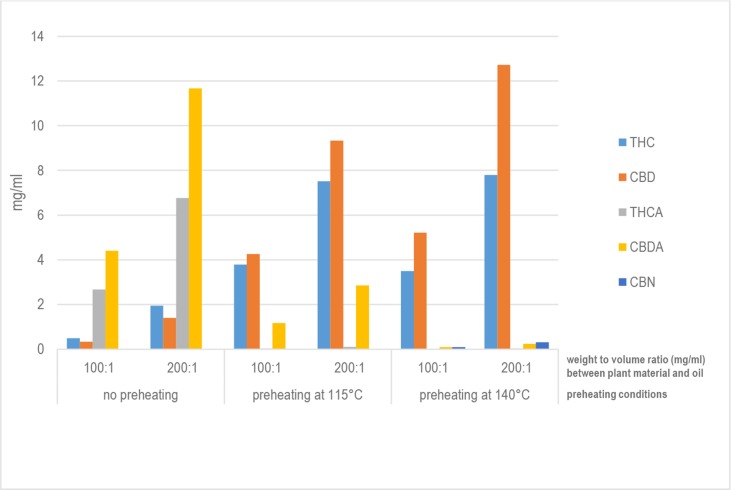
Quantity of active constituents in oils.

**Figure 2:**
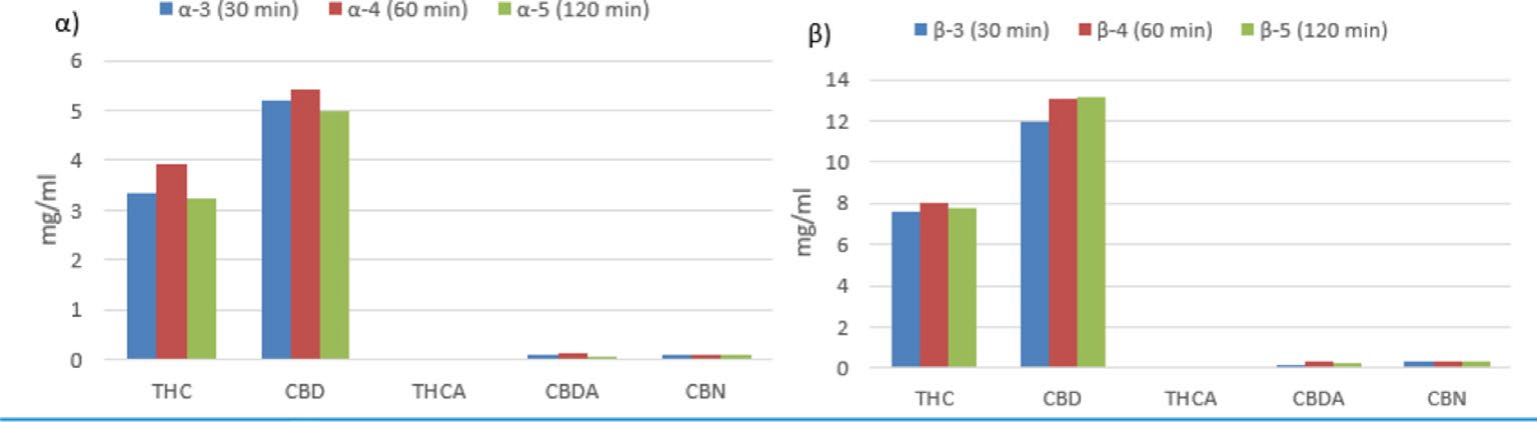
Comparison of the amount of active constituents in oils depending on the length of time in the boiling water bath if weight/volume ratio (mg/ml) between plant material and oil is 100:1 (α) and if it is 200:1 (β).

**Table 4** and **Figure 1** report the quantities of THC, THCA, CBD, CBDA, and CBN detected in 93 samples of oils prepared using the different procedures listed in **Table 2**. Based on the results of the analyses, it can be affirmed that, considering the average content of active constituents, the proportion of acidic forms to decarboxylated forms is greater than 1 only for those methods that do not include a preheating phase for the raw *Cannabis* plant material (α-1 and β-1). As far as the methods that include a preheating phase are concerned, the quantity of active constituents in acid form is proportionally greater for those methods that apply a preheating temperature of 115°C for 40 min (α-2 and β-2) compared to that for methods that apply a preheating temperature of 140°C for 30 min (α-3, α-4, α-5, β-3, β-4, and β-5). Furthermore, the results demonstrate that the length of time in the water bath at 100°C appears to be uninfluential on the quantity of active molecule in the final product. In fact, there were no significant differences in the recovered quantities of THC and CBD in the oils prepared by the methods α-3, α-4, and α-5 with a ratio of *Cannabis* plant weight to solvent volume (mg/ml) of 100:1. The same is true for the oils prepared by the methods β-3, β-4, and β-5 with a ratio of plant weight to solvent volume of 200:1 (**Figure 2**).

**Table 4:** Quantity of active constituent in oils.

Method	Active constituent	Number of samples analyzed	Average concentration of active constituent (mg/ml)	Standard deviation	Concentration of active constituent: minimum value (mg/ml)	Concentration of active constituent: maximum value (mg/ml)
α-1	THC	10	0.49	0.14	0.33	0.71
	CBD		0.33	0.19	0.16	0.67
	THCA		2.67	0.45	2.07	3.29
	CBDA		4.40	0.84	3.12	5.45
	CBN		N.D.	N.D.	N.D.	N.D.
α -2	THC	10	3.79	0.62	3.05	5.09
	CBD		4.26	0.69	3.32	5.45
	THCA		0.03	0.01	0.01	0.05
	CBDA		1.17	0.22	0.82	1.43
	CBN		N.D.	N.D.	N.D.	N.D.
α-3	THC	6	3.35	0.22	3.07	3.65
	CBD		5.22	0.40	4.87	5.75
	THCA		N.D.	N.D.	N.D.	N.D.
	CBDA		0.09	0.03	0.04	0.13
	CBN		0.09	0.04	0.04	0.15
α-4	THC	6	3.92	0.14	3.67	4.09
	CBD		5.44	0.34	4.92	5.94
	THCA		N.D.	N.D.	N.D.	N.D.
	CBDA		0.14	0.03	0.10	0.17
	CBN		0.11	0.01	0.10	0.13
α-5	THC	12	3.22	0.47	2.44	3.88
	CBD		4.98	0.67	4.07	6.58
	THCA		N.D.	N.D.	N.D.	N.D.
	CBDA		0.05	0.02	0.01	0.08
	CBN		0.08	0.02	0.04	0.11
β-1	THC	4	1.94	0.11	1.82	2.09
	CBD		1.40	0.16	1.18	1.55
	THCA		6.76	0.51	6.15	7.40
	CBDA		11.67	0.46	11.21	12.22
	CBN		N.D.	N.D.	N.D.	N.D.
β-2	THC	6	7.52	0.83	6.47	8.68
	CBD		9.33	1.05	8.25	11.12
	THCA		0.11	0.04	0.07	0.17
	CBDA		2.85	0.44	2.36	3.45
	CBN		N.D.	N.D.	N.D.	N.D.
β-3	THC	6	7.59	0.52	6.93	8.30
	CBD		11.96	0.84	10.79	12.89
	THCA		N.D.	N.D.	N.D.	N.D.
	CBDA		0.14	0.02	0.11	0.16
	CBN		0.31	0.06	0.25	0.40
β-4	THC	27	8.04	1.83	5.20	12.19
	CBD		13.05	1.78	9.85	15.58
	THCA		0.03	0.02	0.01	0.07
	CBDA		0.36	0.36	0.11	1.35
	CBN		0.32	0.12	0.13	0.61
β-5	THC	6	7.76	0.66	6.86	8.45
	CBD		13.15	0.94	12.04	14.64
	THCA		N.D.	N.D.	N.D.	N.D.
	CBDA		0.22	0.03	0.18	0.24
	CBN		0.31	0.06	0.24	0.40

In terms of active constituents in the oils produced, it is clear that, for preparations produced under identical conditions (time in boiling water bath, time and temperature of preheating), the proportion of the quantities of THC extracted with methods β compared to the quantities of THC extracted with methods α was, on average, 2.2. For CBD extraction, the same relationship is equal to an average value of 2.4.

CBN, a degradation product of THC, is present in minimal quantities in the samples of oils. CBN was not detected in those samples prepared with methods that do not include preheating of flowering tops (α-1 and β-1) or include preheating at lower temperatures (α-2 and β-2).

## Discussion

The analysis of the decoctions prepared using the different procedures demonstrated that, for a fixed ratio of *Cannabis* plant weight to solvent volume, the decoctions obtained have the same characteristics in terms of concentration of active constituents of interest. Concerning the preparation methods in series B, the objective was to evaluate whether and if so, to what extent, it was possible to enrich the decoctions in terms of cannabinoid content in the decarboxylated form given that these molecules, even though they are easily absorbed in the intestine, not hydrophilic, and, therefore, present in low concentrations in the final decoction. As well as being foreseeable, this could lead to an ineffective therapy. In this context, the results obtained enabled us to observe that changing the ratio of *Cannabis* plant weight to solvent volume, as in methods B-1 to B-3 (ratios from 2:1 to 4:1), did not lead to a significantly greater recovery rate of the active constituents compared to the decoctions prepared using the methods in series A. Regarding methods B-4 and B-5, with proportions of plant weight to solvent volume of 5:1 and 10:1, respectively, the cannabinoid content was greater and, hence, for these decoctions, a greater number of tests were performed. Significantly, when the ratio of plant weight to solvent volume is greater or equal to 4:1, in this study, methods B-3 to B-5, the proportionality between THCA and THC turns out to be in favor of the decarboxylated form despite the use of an aqueous solvent.

Regardless of the fact that increasing the quantity of *Cannabis* plant material used means increasing the quantities of THC and CBD in the decarboxylated form in the final solution, the titrated quantity of the active components in the final decoction is greatly inferior compared to that in the oils. Thus, to ingest the same quantity of active molecule, the patient would have to ingest an extremely high volume of the decoction, with obvious drawbacks. By way of example, considering the most concentrated decoction, which is possible to obtain in terms of CBD (method B-5) and comparing it with the best of the oil preparations (method β-4), it is possible to calculate that the former is approximately 100 times less concentrated compared to the latter; likewise, THC concentration is roughly 60 times less.

As for the oil preparations, based on the results obtained, it is possible to declare that the raw plant preheating phase is fundamental in obtaining oils with the decarboxylated forms of active constituents in proportionally higher concentrations. Therefore, these are more easily assimilated at the intestinal level. Turning to the variations applied to the preheating phase, the best results were observed by applying heat at a higher temperature and for a shorter duration (140°C for 30 min) rather than a lower temperature preheating with a longer duration (115°C for 40 min). With the aim of obtaining higher levels of decarboxylated forms of the active molecules contained in the raw plant, it is fundamental that, during the preheating, the layer of drug has a thickness no greater than 5 mm and ideally no greater than 1–2 mm so that the heat can spread uniformly throughout the layer of plant material.

With regards to the boiling water bath, the results demonstrate that the length of time tends to have no influence on the concentration of constituents in the final product. As a result, it appears clear that it is not necessary to overly extend (120 min) the water bath phase. As a precautionary measure, it is deemed that the recommended method for the preparation of *Cannabis* oils should have a water bath phase with a 60-min duration.

Considering the relationship between raw plant weight to solvent volume and the final concentration of active ingredients in the oils, it is possible to affirm that, by doubling the quantity of plant material used and under identical experimental conditions, the concentrations of THC and CBD in the oils increase by more than twofold. Indeed, the proportion of THC obtained using the methods β and the THC obtained using the methods α increase more than a factor of 2; similarly, for CBD. Therefore, the methods applied in series β led to a more efficient recovery of THC and CBD. Hence, considering this fact and the results regarding the water bath step, it has been established that the most efficient procedure for the preparation of *Cannabis* oils is β-4. An Italian patent application has been lodged with the Italian Office for Patents and Brands for the procedure for *Cannabis* oil production and is currently pending.

All of the preparations obtained in this project were produced by applying standard operating procedures specifically designed for this study. Despite this fact, the oils prepared using the recommended procedure β-4 are those with the highest standard deviation of all the preparations. The variability in the preparations may be linked, in general, to the fact that the vegetable matrix itself is not completely standardizable and, as is known, is affected by variations over time in the content of active ingredient.

The extractive capability of oil for cannabinoids was better than the water one. This was predictable because the cannabinoids have a lipophilic nature. The decoctions were prepared and analyzed because they are right now a frequent form of administration and recommended by the Italian Ministry of Health.

New formulations based on *Cannabis* oil are increasingly used. For instance, a study demonstrated that adding medical *Cannabis* oil to Alzheimer’s disease patients’ pharmacotherapy is safe and a promising treatment option in relieving behavioral and psychological symptoms of dementia (Shelef et al., 2016). Similarly, a prospective, open-label trial in children with Dravet syndrome demonstrated that a meaningful clinical reduction in seizures rates is achievable in pharmacoresistant participants by the addition of *Cannabis* oil to their concomitant antiepileptic regimens (McCoy et al., 2018). However the problem of oil standardization still needs to be addressed, as also recently documented by a study showing that adolescents and young adults with inflammatory bowel disease used *Cannabis* oil with a variety of delivery methods and concentrations and ratios of CBD to THC (Hoffenberg et al., 2019).

## Conclusions

Based on the results obtained, it is possible to state that prescribing *Cannabis*-based decoctions, considering the low recovery rates of THC and CBD and, consequently, the high volume of preparation that the patient would have to ingest, should not be the first choice for *Cannabis*-based therapies, as is, unfortunately, currently recommended by the Italian Ministry of Health. Moreover, it is opportune to take into account the high raw material costs required to obtain the desired quantities of active molecules as previously discussed. In fact, to administer a similar dose of THC and CBD, it would be necessary to utilize a considerably higher quantity of *Cannabis* to prepare a decoction compared to that required to prepare an oil-based preparation.

As for the oil preparations, procedure β-4 proved to be the most efficient method, with average recovery rates of 8.04 mg/ml of THC and 13.05 mg/ml of CBD, both in decarboxylated forms. These values are significantly higher compared to those with water-based extraction (decoctions) or other current oil-based extraction techniques.

Despite the excellent results achieved for the oils in terms of recovered active constituent concentrations, a consequence of the application of strict operating protocols, it is not possible to do away with the titration procedure for the oils, also taking into account that this is currently a requirement under Italian regulations.

At present, a study on the stability of the formulations, both in decoction and in oil forms, produced using the procedures developed as part of this research is being carried out.

Considering that the oils have a major drawback in that they have an unpleasant taste and smell, which may affect adherence to the therapy and hinder the use of a placebo arm in clinical research, the development of a pharmaceutical-grade formulation for oral administration is underway, exploiting the oil formulations to facilitate patients prescribed *Cannabis*-based medical therapies.

## Patent

An Italian patent application has been lodged with the Italian Office for Patents and Brands for the procedure for *Cannabis* oil production and is currently pending.

## Data Availability Statement

All datasets generated for this study are included in the manuscript and the supplementary files.

## Author Contributions

FB and PB performed the conceptualization of the work. FB and IP performed the investigation, developed the methodology, and took care of the data. MS performed the analytical data acquisition. LRE performed statistical analysis. FB wrote the manuscript. RT, ADL, MC, and AD’A supervised the project. PB coordinated and administered the project. All authors approved the final version of the study.

## Funding

The study was supported by the fund “Fondi Ricerca Locale (ex 60%)” of the University of Turin provided by the MIUR (Ministry of Education, Universities and Research).

## Conflict of Interest Statement

The authors declare that they have no known competing financial interests or personal relationships that could have appeared to influence the work reported in this paper.

## Abbreviations

THCA, delta-9-tetrahydrocannabinolic acid; CBDA, cannabidiolic acid; THC, delta-9-tetrahydrocannabinol; CBD, cannabidiol; CBN, cannabinol; CE, collision energies
